# SEEG‐based reevaluation of epileptogenic networks and the predictive role for reoperation in MTLE patients with surgical failure

**DOI:** 10.1002/epi4.12743

**Published:** 2023-04-27

**Authors:** Ke Xu, Xue Yang, Jian Zhou, Yuguang Guan, Meng Zhao, Mengyang Wang, Jing Wang, Tianfu Li, Xiongfei Wang, Guoming Luan

**Affiliations:** ^1^ Department of Neurosurgery, Sanbo Brain Hospital Capital Medical University Beijing China; ^2^ Department of Neurology, Sanbo Brain Hospital Capital Medical University Beijing China; ^3^ Department of Brain Institute, Center of Epilepsy, Beijing Institute for Brain Disorders, Beijing Key Laboratory of Epilepsy Research, Sanbo Brain Hospital Capital Medical University Beijing China

**Keywords:** epileptogenic network, functional connectivity, mesial temporal lobe epilepsy, reoperative outcomes, SEEG reevaluation

## Abstract

**Objective:**

Approximately 20%–30% of mesial temporal lobe epilepsy (MTLE) patients got unfavorable seizure control after surgery, and there was a discrepancy about the reasons for the surgical failure. The functional connectivity (FC) patterns obtained from stereo‐electroencephalography (SEEG) reveal information about the dynamics of the epileptic brain and the added value of extracting information that was not identifiable in the SEEG data using FC analysis. This study aims to find out the patterns of the potential epileptogenic network of failure patients and the electrophysiological predictors of reoperation.

**Methods:**

From January 2012 to December 2019, the MTLE patients with surgical failure were reviewed, and all patients underwent SEEG‐guided reoperation. The epileptogenic network was quantified by calculating FC indicators, including phase slope index (PSI), mutual information (MI) strength, imaginary coherence (icoh), and Granger causality.

**Results:**

Ten patients with 13 seizures were included in the analysis, and 7 of them achieved a favorable outcome after the SEEG‐guided reoperation. The surgical zone (SZ) with a favorable prognosis showed greater outward information flow than the non‐SZ, whereas the SZ with an unfavorable prognosis showed greater inward information flow. The recurrent patients with favorable prognosis had strong connectivity between the posterior hippocampus, temporal neocortex, and insula, whereas the patients with unfavorable prognosis showed strong functional connectivity between the insula and temporal–parietal‐occipital junction. The power spectrum of patients with favorable prognosis was significantly lower than that of patients with unfavorable prognosis, especially showing a more oscillation power of low frequency.

**Significance:**

The SEEG‐guided reoperation could achieve favorable seizure control outcomes for recurrent patients. The FCs were a potential indicator to help construct the temporal epileptic network and predictor for the reoperative prognosis in the recurrent patients.

**Key points**

- SEEG‐guided reoperation could achieve favorable seizure control outcomes for patients with surgical failure.
- Differences in the epileptic network between recurrent patients with favorable and unfavorable outcomes.
- FC was the predictor for the reoperative prognosis in the recurrent patients.

## 1 INTRODUCTION

Mesial temporal lobe epilepsy (MTLE) is one of the most common forms of intractable epilepsy.^1^ Standard anterior temporal lobectomy (ATL) is an established effective surgery in MTLE patients.^2^, ^3^ However, ATL surgery still fails to provide a seizure‐free outcome in 20%–30% of these patients.^4^ The analysis of surgical failure was also controversial. Salanova et al.^5^ reported that the remaining hippocampus caused the ATL failure by the further resection of the mesial temporal structure alone, with 64% achieving a seizure‐free outcome. However, Barba et al.^6^ demonstrated that temporal plus epilepsy (TPE) was a major determinant of temporal lobe surgery failures by the retrospective analysis of preoperative stereo‐electroencephalography (SEEG). Besides, the low seizure‐free rate of reoperation had been reported before.^7^, ^8^, ^9^ The criteria for the reevaluation were significant for the different seizure‐free rates from the previous studies.^9^, ^10^ Therefore, it is necessary to find and reevaluate biomarkers to improve the effect of reoperation.

The correlation between brain regions called functional connectivity (FC), allows us to identify the specific network that is functionally connected.^11^ The FC patterns obtained from SEEG reveal information about the dynamics of the epileptic brain that can be used to predict an upcoming seizure and to localize the seizure onset zone (SOZ). The added value of extracting information that is not identifiable in the SEEG data using FC analysis is stressed.^11^ This network analysis method may supplement clinical interpretation by defining epileptogenic networks without requiring ictal recordings, and methods are currently being explored.^12^, ^13^, ^14^ In the SEEG‐FC analysis, the connectivity between brain regions is quantifiable by a multitude of measures dependent on the type of network features one wishes to investigate. The nondirected FC calculation, such as mutual information (MI) and imaginary coherence (icoh), can be used to quantify the number of interactions between neural populations^15^; and the directed FC calculation, such as Granger causality and phase slope index (PSI) can be used to estimate the direction of information flux in multivariate time series and distinguished the driver from the recipient.^16^ These measures have been utilized in previous MTLE studies to estimate the FC network with different epileptic periods or hemispherical laterality.^17^, ^18^ However, few studies explore the MTLE network through SEEG‐FC evaluation of patients with ATL failure. Therefore, this study aimed to explore the underlying epileptic network of MTLE patients with surgical failure by SEEG‐FC analysis from the level of inner brain region (contacts), brain regions, and the SOZs respectively. A second objective is to evaluate the predictive effect of the epileptic network on the reoperation.

## 2 METHODS

### 2.1 Patients selection

We retrospectively reviewed the patients with failed ATL from January 2012 to December 2019 in the Department of Neurosurgery at Sanbo Brain Hospital, Capital Medical University. All recruited patients experienced unfavorable seizure outcomes after ATL, and the implantation of SEEG electrodes was performed to reevaluate the SOZ for them. Consequently, individual preoperative plans, including radiofrequency thermocoagulation, the resection of the SOZ, and the bipolar electro‐coagulation of the functional cortex were determined by the SEEG results.

The exclusion criteria were the following: (a) patients with nonstandard ATL, (b) failed patients who underwent reoperation directly, (c) patients who underwent vagus nerve stimulation (VNS), and (d) patients who were followed up for less than 12 months after reoperation. This study was approved by the Ethics Committee of Sanbo Brain Hospital, Capital Medical University (SBNK‐2017‐15‐01).

### 2.2 Anterior temporal lobectomy and seizure outcomes

Criteria to perform primary surgery were the following: (a) typical seizure aura or semiology of the temporal lobe, (b) MRI signs with mesial temporal sclerosis, (c) interictal and ictal electroclinical findings of long‐term video‐electroencephalography (EEG) judged typical for unilateral MTLE. The standard surgical process included the resection of the anterolateral temporal lobe tissue (3.5 cm of dominant side; 4.5 cm of nondominant side), a complete amygdalectomy, and the anterior part of the hippocampus before the posterior boundary of the brainstem.^2^ The seizure outcomes were evaluated at 3 months postoperatively and yearly thereafter. Engel classification system^19^ was used for seizure outcomes assessment, and Engel classes II–IV were defined as surgical failure.

### 2.3 Preoperative evaluation

The preoperative variables were collected from the medical records, which included recurrent time, postoperative time, aura, seizure type, MRI examinations, video‐EEG, magnetoencephalography (MEG), [^18^F]‐fluorodeoxyglucose positron emission tomography (^18^FDG‐PET), and stere‐oelectroencephalography (SEEG). The brain MRI of recurrent patients was scanned with a 1.5 scanner for T1, T2, and T2 fluid‐attenuated inversion recovery (FLAIR) sequences. The standard 64‐channel long‐term video‐EEG monitoring was used in patients for at least 24 hours. The video‐EEG was sampled at the rate of 1024 samples and recorded in a double banana montage. Besides, the MEG was used to delineate the SOZ by localizing interictal epileptic spikes, and the PET was used to locate the hypometabolic regions. After completion of the presurgical evaluation by neurosurgeons, neurologists, neuropsychologists, electrophysiologists, and neuroradiologists, the decision of SEEG implantation was made.

### 2.4 The approach of SEEG implantation

The implantation regions of SEEG electrodes were based on noninvasive findings. The major regions included the residual hippocampus (pHip), middle temporal gyrus (MTG), posterior superior temporal gyrus (pSTG), and extratemporal regions (insular cortex, temporal–parietal–occipital [TPO], and orbitofrontal gyrus [OFG]). Accordingly, the protocol of SEEG implantation for ATL failure was proposed, and the following electrodes were indispensable: (a) from the pHip to the MTG, (b) from the insula to the transverse temporal gyrus to the pSTG, (c) from the OFG to the anterior cingulate gyrus to the superior frontal gyrus, (d) from the posterior cingulate gyrus to the supramarginal gyrus or the angular gyrus, and (d) from the insular long gyrus (ILG) to the insular short gyrus (ISG) to the middle frontal gyrus (oblique insertion). Besides, the contralateral hemisphere and other highly suspicious regions of individuals would increase the coverage accordingly. Each electrode had a diameter of 0.8 mm and consisted of 5–18 contacts 2 mm in length with 1.5 mm spacing, (Beijing HKHS Healthcare Co., Ltd, Alcis). Three‐dimensional T1‐weighted double‐enhanced contrast MRI was performed to avoid the injury of cranial major vessels. Electrodes were implanted using the Robot of Surgery Assistant (ROSA) system under general anesthesia. The reconstruction of postoperative computed tomography (CT) and preoperative MRI was performed on the Brainstorm toolbox (https://neuroimage.usc.edu/bst/download.php) of MATLAB software (R2017a) to confirm the position of SEEG electrodes. The SEEG data were recorded using a reference electrode (Nicolet™ system; 128 channels; sampling rate: 512 Hz). At least twice habitual seizures were caught during the SEEG monitoring unit.

### 2.5 SEEG analysis

The details of the SEEG analysis were presented in the Appendix S1.

### 2.6 Reoperation planning and follow‐up

The reoperation planning was as follows: (a) for patients with epileptogenic discharge from the posterior hippocampus or insular cortex: the SEEG‐guided radiofrequency thermocoagulation (RFTC) was performed preliminarily to reduce the neurofunctional impartment. The RFTC procedure was delivered by the power to a maximum level of 3.5 W and maintained the power level for 2 minutes. Thereafter, the epileptogenic zone resection was performed for those unfavorable seizure outcome patients after RFTC; (b) for the propagation region involving the insular cortex, bipolar electro‐coagulation of the insular cortex with epileptogenic zone resection was planned to reduce vascular injury. Bipolar electro‐coagulation of the functional cortex (BCFC) was performed by the bipolar electro‐coagulation with 3–5 W output power. The insular surface was kept clean and moist with saline gauze before BCFC was performed. There was a 45° angle between the forceps axis and the insular surface. Keeping the electro‐coagulation perpendicular to the long axis of the insular gyrus, the tip diameter of the bipolar forceps was 2 mm. The stimulation was applied with a 1 second duration, and the insular surface was rinsed with saline to lower the temperature immediately after electro‐coagulation.^20^ BCFC was similar to subpial transection multiple (MST), with bipolar coagulation being the replacement for MST.^20^ The resection of the epileptogenic zone was strictly according to the presurgical plan, and the postoperative MRI was taken to confirm. The seizure outcome was recorded from the second postoperative year and assessed by neurosurgeons in the outpatient clinic or by telephone interview according to the Engel classification.^21^ The follow‐up was more than 24 months, and Engel class I was classified as a favorable seizure outcome (seven patients: group one), and Engel classes II–IV were classified as an unfavorable seizure outcome (three patients: group two).

### 2.7 Statistical analysis

Continuous variables were described using means ± standard deviation (SD). The Shapiro–Wilk test was used to determine if the FC values were normally distributed. The Mann–Whitney *U* test was used to compare FC values between the surgical zone (SZ: RFTC, BCFC, or resected zone) and non‐SZ of the two groups, respectively. Besides, the Mann–Whitney *U* test was used to compare the difference between the two groups per brain region. All comparisons were produced involving seven frequency bands. The accuracy discrimination between SZ and uninvolved zones using FC measures included MI strength, icoh, Granger causality strength, and PSI. The permutation test (Fieldtrip Toolbox, 5000 times, adjusted alpha level = 0.01) was used to compare the difference in power spectra distribution between the two groups. All statistical analysis was performed with Prism 8.0, and a *P*‐value < .05 was considered significant.

## 3 RESULTS

### 3.1 Patients characteristics

A total of 10 failed patients (six male and four female) who underwent SEEG implantation were evaluated in this study. The mean recurrent time after ATL was 8.4 ± 7.26 years, and the mean time of postoperation was 9.9 ± 5.66 years. Three patients (30%) had an aura, and 6 patients (60%) had secondarily generalized tonic–clonic seizures (sGTCS). During the video‐EEG monitoring, the ictal epileptic discharge was recorded in five patients (50%) arising at the unilateral temporal lobe; in three patients (30%) arising at the unilateral hemisphere; in two patients (20%) arising in the bilateral hemisphere. The MEG spikes sources were observed in eight patients (three patients were unilateral temporal lobe, three patients were unilateral multiple lobes, one patient was unilateral insula, and one patient was bilateral temporal lobes), and two patients showed a negative result. Besides, the hypometabolic regions of PET were observed in all patients (two patients were unilateral temporal lobe, six patients were unilateral hemisphere, two patients were bilateral hemisphere, Table 1). Thereafter, the suspicious SOZs from the results of video‐EEG, MEG, and PET were evaluated for SEEG implantation.

**TABLE. 1:** Demographic and preoperative evaluation of recurrent patients at the second admission.

Case No.	Recurrent time (year)	Postoperative time (year)	Aura	Seizure type	ID on VEEG	MEG findings	PET
1	7	9	None	Tonic–clonic	R. temporal	R. temporal and parietal	R. hemisphere
2	1	4	None	Tonic–clonic	R. temporal	R. Insular	R. hemisphere
3	6	7	Déjà vu	Absence	L. temporal	L. temporal	Bilateral temporal
4	5	6	None	Absence and Automatism	R. temporal	R. temporal	R. temporal & parietal
5	5	7	None	Absence and Heart palpitations and Automatism	L. hemisphere	Negative	L. temporal
6	13	16	None	Absence and Automatism and Tonic–clonic	Bilateral diffuse	L. temporal	L. temporal
7	15	17	None	Heart palpitations and Tonic–clonic	R. temporal	R. temporal and insula and parietal	R. hemisphere
8	20	26	None	R. tonic seizure	L. hemisphere	L. temporal & parietal	L. hemisphere
9	1	2	Headache	Tonic–clonic	R. hemisphere	Bilateral temporal	R. hemisphere
10	5	11	Rising epigastric sensation	Absence and Automatism and Tonic–clonic	Bilateral diffuse	Negative	Bilateral hemisphere

Abbreviations: ID, ictal discharge; L, left; MEG, magnetoencephalography; PET, positron emission tomography; R, right; VEEG, Video‐Electroencephalography.

### 3.2 SEEG results and reoperation

Ten patients underwent unilateral SEEG implantation based on the reevaluation, and one patient underwent bilateral SEEG implantation because of the unclear SOZs side. As the SEEG results showed, the SOZs of three patients (30%) were from the remnant hippocampus; one patient (10%) was from the MTG, and six patients (60%) were from the temporal neighboring regions (five ILG and one OFG). Furthermore, the insular cortex was involved in the early propagation zone in nine patients (WHO, Table 2).

**TABLE. 2:** SEEG analysis and reoperation plan data for MTLEs with surgical failures.

Case no.	No. of SEEG (L:R)	SOZs confirmed by SEEG results	RFTC	Resection region	BCFC	Engel class (2 years after reoperation)	Pathology
1	0:11	Second ILG → ISG + posterior cingulate gyrus	ILG	TTG	ILG	III	FCD I b
2	0:9	Second ILG → ISG + medial OFG	ILG	STG	ILG	II	Pathological changes after brain injury
3	9:0	Hippocampal tail → posterior temporal cortex + TPO	Posterior hippocampus	Residual hippocampus and MTG	None	I	Pathological changes after brain injury
4	0:9	Posterior MTG → first ILG + posterior temporal cortex	None	MTG and residual hippocampus	ILG	I	Pathological changes after brain injury
5	12:0	Posterior hippocampal body + Hippocampal tail → ISG	Posterior hippocampus	Residual hippocampus and STG	ISG	I	Severe HS
6	9:0	First ILG → first ISG + frontal operculum	ILG	None	None	I	None
7	0:13	Second ILG → first ILG + TPO + middle cingulate gyrus	ILG	STG	ILG	III	FCD II a
8	10:1	Second ILG → first ILG + supramarginal gyrus + posterior cingulate gyrus	ILG	STG	ILG	I	Pathological changes after brain injury
9	0:14	Medial OFG → ISG + anterior cingulate gyrus	None	Frontal lobectomy	ISG	I	FCD III d
10	6:3	Posterior hippocampal body → ISG + OFG	Posterior hippocampus	Residual hippocampus and MTG	ISG	I	Mild HS and Pathological changes after brain injury

Abbreviations: BCFC, bipolar electro‐coagulation of functional cortex; FCD, focal cortex dysplasia; GMH, gray matter heterotopia; HS, hippocampal sclerosis; ILG, insular long gyrus; ISG, insular short gyrus; L, left; MTG, middle temporal gyrus; OFG, orbital frontal gyrus; R, right; RFTC, radiofrequency thermocoagulation; SEEG, stereo‐electroencephalography; SOZ, seizure onset zone; STG, superior temporal gyrus; TPO, temporal–parietal–occipital; TTG, transverse temporal gyrus.

Eight patients originating from the remnant hippocampus and insula gyrus underwent RFTC first, and one of them achieved a seizure‐free outcome (Patient 6). Besides, nine patients underwent resection of the EZ. The BCFC combined with resection (eight patients, 80%) was performed for those patients who involved insular propagation, and only two patients had postoperative transient aphasia but recovered in 3–5 days. Surgical specimens of nine patients were processed for histological analysis as a routine. Three patients (30%) were diagnosed with focal cortical dysplasia (FCD I b: one patient; FCD II a: one patient; FCD III d: one patient); one patient (11.11%) was diagnosed with severe hippocampal sclerosis (HS); four patients (44.44%) were diagnosed with pathological changes after brain injury; one patient (11.11%) was diagnosed with mild HS combined with pathological changes after brain injury. Finally, seven patients (70%) were achieved seizure‐free in the 2 years after reoperation (group one), and three patients still got unfavorable seizure outcomes (group two).

### 3.3 Comparison of FCs between SZ and non‐SZ

**FIGURE 1:**
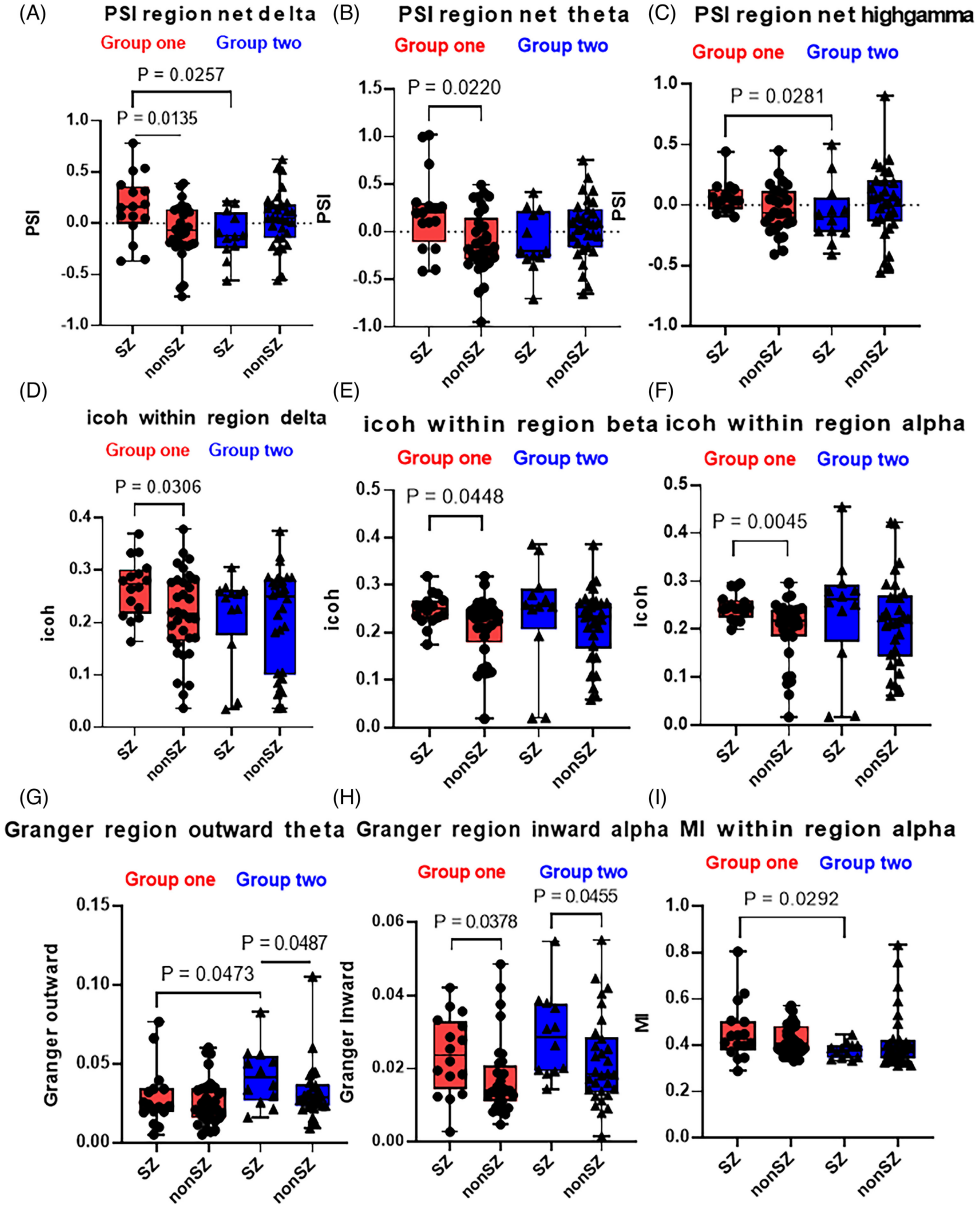
The comparison of FCs in SZ and non‐SZ between recurrent patients with favorable and unfavorable outcomes. The SZ of group one and non‐SZ of group two had net outward information flux (positive PSI), whereas the non‐SZ of group one and SZ of group two suggested a net inward information flux (negative PSI; A–C). The icoh values of SZ and non‐SZ were significantly different in group one (D–F). The SZ of the two groups both showed significantly higher Granger outward information flow and inward information flow than non‐SZ. The SZ of group two showed lower Granger flux and MI than the SZ of group one, especially in the lower frequency band (G–I). PSI, Phase Slope Index.

Across the full frequency bands, the SZ of group one had net outward information flux (positive PSI), whereas the SZ of group two and non‐SZ of group one had a net inward information flux (negative PSI; Figure 1A–C). The PSI values of SZ in group one were significantly higher than that of non‐SZ in group one within the delta and theta frequency band (*P* = 0.014; *P* = 0.022; Figure 1A–C).

**FIGURE 2:**
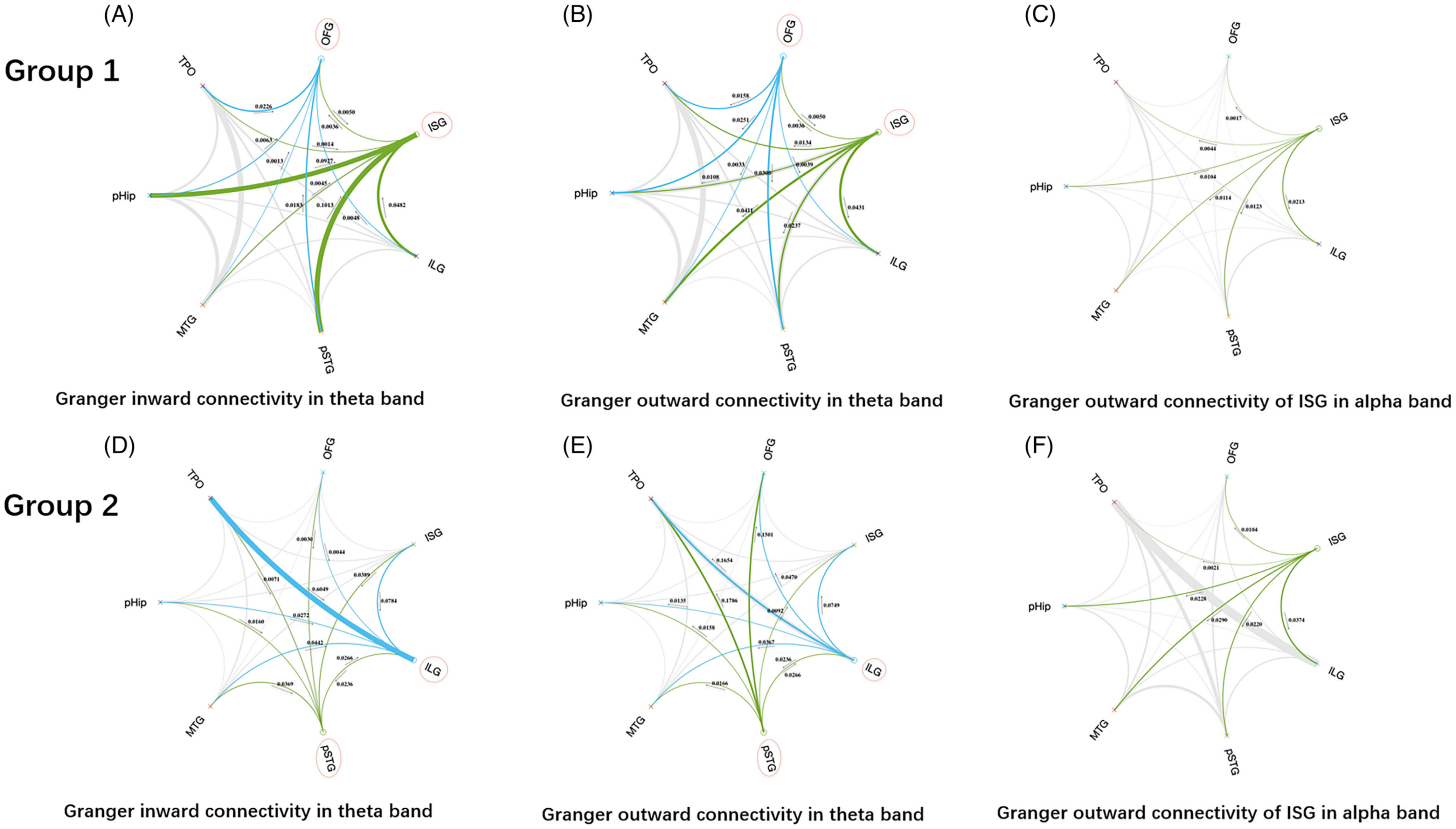
The differences in Granger causality in SZ between two groups. The Granger causality of SZ showed higher outflow connectivity than other regions in group one theta band (A, B, D, E). The Granger causality of ISG in group two showed higher outward flow than that in group one in the alpha band (C, F). ILG, insular long gyrus; ISG, insular short gyrus; MTG, middle temporal gyrus; OFG, orbital frontal gyrus; pHiP, posterior hippocampus; pSTG, post‐superior temporal gyrus; TPO, temporal–parietal–occipital.

**FIGURE 3:**
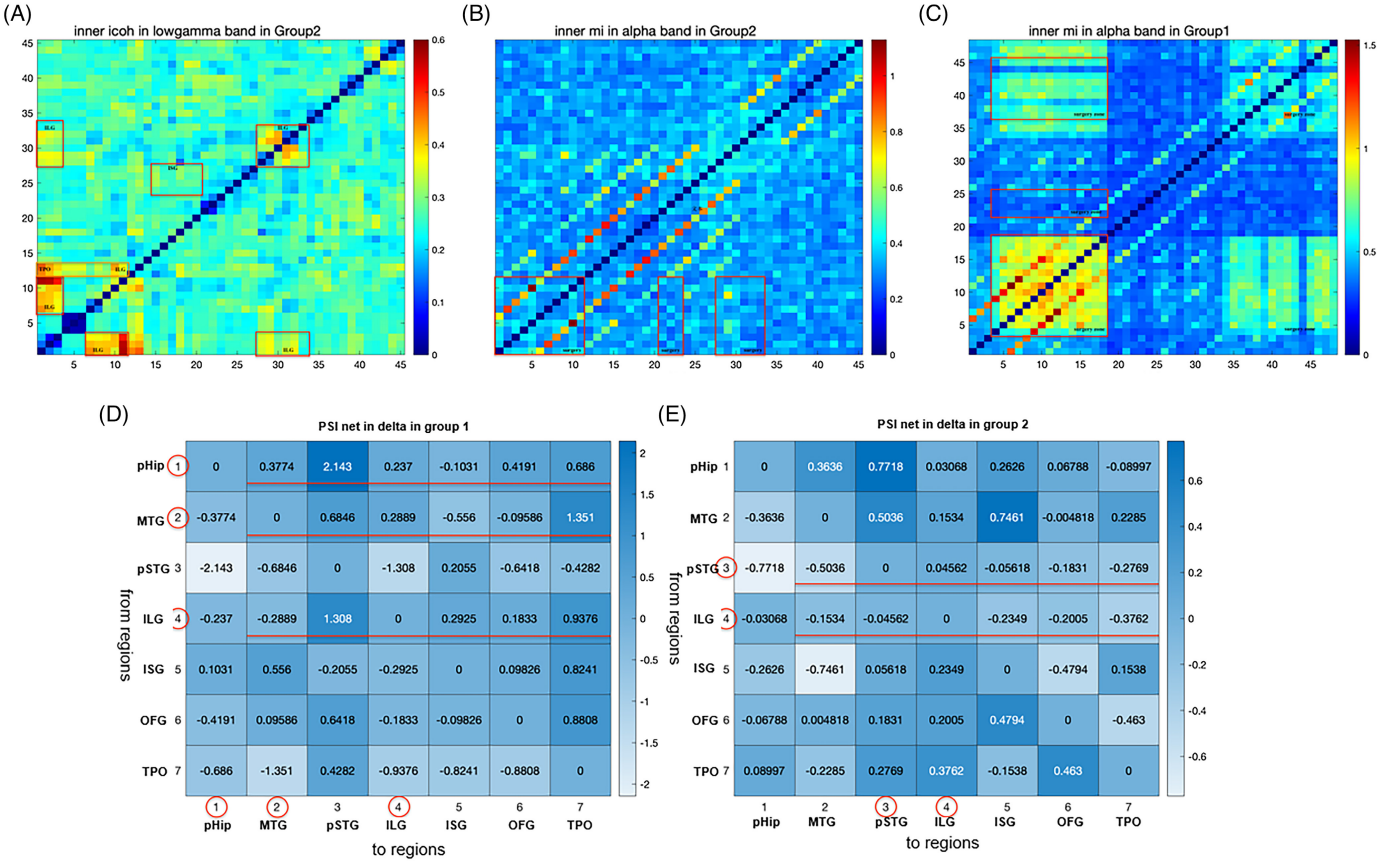
The differences in FC matrics, including inner icoh, MI strength, and PSI between the two groups. The icoh of ILG regions showed significantly higher than that of other regions in low‐gamma bands (A). The MI strength of SZ in group one showed significantly higher than that of SZ in group two in the alpha band (B, C). The SZ of group one had a net outward information flux (positive PSI), while group two suggested a net inward information flux (negative PSI; D, E). ILG, insular long gyrus; ISG, insular short gyrus; MTG, middle temporal gyrus; OFG, orbital frontal gyrus; pHiP, posterior hippocampus; pSTG, post‐superior temporal gyrus; TPO, temporal–parietal–occipital.

The SZ showed significantly higher in both Granger outward information flow and inward information flow than non‐SZ in all recurrent patients (Figure 1G,H). Besides, the Granger mutual information of SZ in group two showed significantly higher than that of SZ in group one (Figure 2). Considering the synchronization of contacts within the same region, the icoh and MI strength of SZ showed significantly higher than that of non‐SZ in group one in delta, alpha, and beta (1–4 Hz, 8–30 Hz) bands (*P* = 0.031; *P* = 0.048; *P* = 0.005; *P* = 0.029; Figures 1D–F,I and 3A–C). However, there was no significant difference in FCs in group two.

### 3.4 Comparison of FCs in brain regions between two groups

**FIGURE 4:**
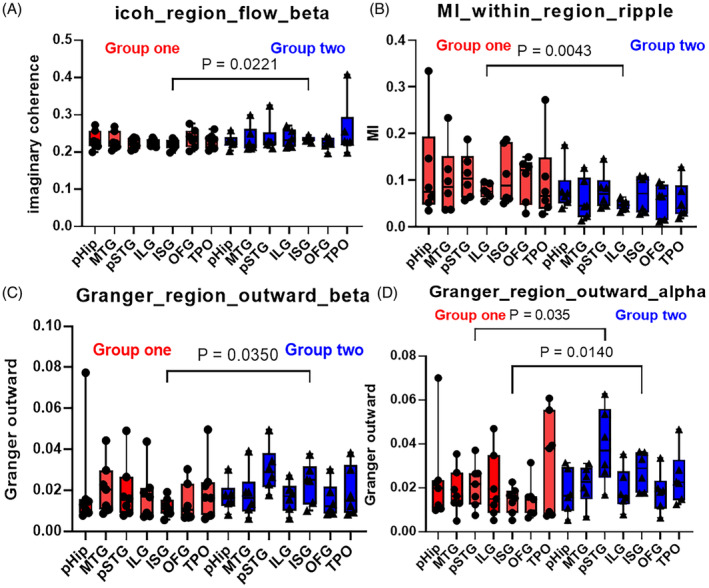
The comparison of FCs in brain regions between two groups. The icoh of the ISG region in group two showed higher than that of group one in the beta band (A). Besides, the ILG's MI strength in group two was significantly lower than that of group one in the ripple band (B). The Granger causality of ISG in group one showed significantly lower than that of group two (C, D). ILG, insular long gyrus; ISG, insular short gyrus; MTG, middle temporal gyrus; OFG, orbital frontal gyrus; pHiP, posterior hippocampus; pSTG, post‐superior temporal gyrus; TPO, temporal–parietal–occipital.

The icoh of ISG in group two showed significantly higher than that of group one in the beta band (*P* = 0.022; Figure 4), and the MI strength of the ILG region in group two showed significantly lower than that of group one in the ripple and alpha bands (*P* = 0.004; Figure 4). Besides, the Granger outward strength of ISG in group two was significantly higher than that of group one in the beta and alpha bands (*P* = 0.035; *P* = 0.014; Figure 4). There were no significant differences in FCs in other regions between the two groups.

### 3.5 Different frequency power spectrum between two groups

The SEEG signals of the two groups were merged to produce frequency transformation to show the distribution of the power spectrum in general (Figure S1). The power spectrum along the frequency axis (full frequency range set was from 1 Hz to 110 Hz) was mainly centralized on the relatively lower frequency bands. Besides, the power spectrum (amplitude^2^) of group two was higher than that of group one, although there was no statistical difference between the two groups by using the permutation test (Figure S1).

## 4 DISCUSSION

In this study, we described a small but detailed cohort of failed ATL patients who underwent SEEG implantation to reevaluate the reasons for ATL failure. The FC analysis of SEEG showed a dramatic difference in the epileptogenic network between reoperative MTLE patients with favorable and unfavorable outcomes and its predictive role for the reoperative prognosis. The SZ had higher information flow strength than non‐SZ in MTLE patients with surgical failure, including Granger outward and inward strength, icoh, and MI strength. The surgical zone (SZ) of group one showed greater outward information flow than the non‐SZ, whereas the SZ of group two showed greater inward information flow. The recurrent patients with favorable prognosis had a strong connectivity between the posterior hippocampus, temporal neocortex, and insula, whereas the patients with unfavorable prognosis showed strong functional connectivity between insula and TPO. The power spectrum of patients with favorable prognosis was significantly lower than that of patients with unfavorable prognosis, especially showing a more oscillation power of low frequency. Therefore, the SEEG implantation was necessary to identify the epileptogenic pattern of MTLE and improve the surgical results.

Recent evidence indicated that seizures most commonly arise from abnormal brain networks rather than isolated focal lesions.^22^, ^23^ Therefore, it is important to identify brain network abnormalities in MTLE in order to accurately map seizure generation.^13^ Our results explored two different FC network patterns in MTLE patients with surgical failure. As the result showed, the recurrent patients with favorable outcomes had a strong connectivity between intratemporal and extratemporal regions (posterior hippocampus‐temporal neocortex‐insula), whereas the patients with unfavorable outcomes showed strong functional connectivity between extratemporal regions (insula‐TPO). This indicated that the reoperation confined to the temporal lobe could not be enough to break through the hemispheric network and cause the surgical failure. Besides, we further explored the differences in the FC matrix between prognoses at the brain region level. As a result, there was no significant difference in inner icoh matrixes between SZ and non‐SZ in group two. Considering the icoh measures were insensitive to signals from a common underlying source, this index could ignore the correlation of sources with a minor time lag. Under such a strict evaluation, it seemed that the highest synchronization among neural populations in ISG. Moreover, a significant difference in the MI strength of SZ between group one (higher) and group two (lower) was observed. These results represented intercontact information communication within SZ. Moreover, the SZ of group two showed more net information inflow than that of group one, which seemed that there was a strong inflow inhibition from peripheral regions to SZ.

According to the differences in frequency power spectrum in the two groups, the patients with unfavorable outcomes had higher power in the low‐frequency band, and the seizure onset pattern of the low‐frequency band was associated with a poorer prognosis. The previous study suggested that the seizure onset pattern of low‐voltage fast activity (LVAF: oscillation frequency > 13 Hz) was more relevant to FCD and better prognosis.^24^ In addition, there was lower MI strength in the ripple band in ILG inner region and higher Granger outward strength in the alpha band from the ISG region to other regions in group two. It further verified that lower functional oscillation was observed in the high‐frequency band in the patients with unfavorable outcomes.

The SZ had higher information flow strength than non‐SZ in recurrent patients, which were consistent with previous studies.^25^, ^26^ Actually, the connectivity studies had reported that normal physiologic networks could be disrupted in MTLE patients.^12^ Besides, the SZ with favorable prognosis showed greater outward information flow than the non‐SZ, whereas the SZ with unfavorable prognosis showed greater inward information flow. The SZ of patients with unfavorable outcomes had higher Granger outward and inward strength than that in patients with favorable outcomes. An SEEG‐FC analysis comparing MTLE patients and the “control” group showed the enhanced connectivity of the epleptogenic zone in the MTLE patients.^27^ Our results confirmed this view that the clarified SOZ by excision had higher outward connectivity, whereas the regions with stronger inward information flow would not be SOZ. Besides, the ILG and ISG inner regions had higher synchronization of neural populations (high icoh within the region) in patients with surgical failure. Conclusively, MTLE patients with higher outward information in the insula had a complex network, which could influence the prognosis of reoperation in recurrent patients.

With the development of advanced neurosurgical techniques, insular epilepsy surgery was proven safe and effective.^28^ In our study, one of three patients with insular discharge achieved seizure‐free after SEEG‐guided RFTC. The efficacy of RFTC was attributed to the extent of the epileptogenic zone covered by SEEG electrodes and the application of optimized parameters. A recent study^29^ developed an optimally extended RFTC lesion guided by cross‐bond SEEG electrodes and confirmed the efficacy for MTLE patients with hippocampal sclerosis. Not as RFTC, the mechanism of BCFC was to block the horizontal conduction of transverse fibers in the three layers of the superficial cortex to prevent the spread of epilepsy.^30^ Ding et al.^31^ reported that bipolar electro‐coagulation with cortextomy for the treatment of insular epilepsy guided by SEEG could achieve favorable seizure control with low neurofunctional deficits. Our result showed that among the 9 patients who underwent the epileptogenic zone resection combined with BCFC for the insular cortex, five patients got seizure‐free after surgery. Therefore, it was feasible to use BCFC to treat epilepsy in which the insular cortex was involved in rapid propagation.

Several limitations restricted the scientific value of this study. Most failed patients were worried about the effect of the reoperation and reluctant to take reoperation. Therefore, a limited number of failed patients were recruited for the analysis. The previous studies^32^, ^33^, ^34^ on the etiology and outcomes of ATL in adults and children were different, but this study did not distinguish between them. In conclusion, further studies on a large scale were necessary to provide better evidence for the MTLE epileptogenic network.

This study was based on the SEEG‐FC to analyze the epileptic network and its relationship with reoperative prognosis in recurrent MTLE patients. The SZ with favorable prognosis showed greater outward information flow than the non‐SZ, while the SZ with unfavorable prognosis showed greater inward information flow. The recurrent patients with favorable prognosis had a strong connectivity between the posterior hippocampus, temporal neocortex, and insula, whereas the recurrent patients with unfavorable prognosis showed strong functional connectivity between extratemporal regions. The power spectrum of patients with favorable prognosis was significantly lower than that of patients with unfavorable prognosis, especially showing a more oscillation power of low frequency. The SEEG‐based FC analysis by evaluating the strength and direction of information flow in the suspected regions was a potential indicator to construct the temporal epileptic network and predict the reoperative prognosis. In conclusion, this study helped us better understand the causes of surgical failure and provide a new direction for more precise network disruption in the further.

## AUTHOR CONTRIBUTIONS

Ke Xu: Formal analysis, Investigation, Writing the original draft. Xue Yang: Investigation, Validation. Jian Zhou: Visualization. Yuguang Guan: Data analysis. Meng Zhao: Validation. Mengyang Wang: Methodology. Jing Wang: Investigation. Tianfu Li: Software, Validation. Xiongfei Wang: Resources, Funding acquisition. Guoming Luan: Conceptualization, Supervision, Writing—review and editing.

## FUNDING INFORMATION

This work was supported by the National Natural Science Foundation of China (81790654, 81790650) and Capital's Funds for Health Improvement and Research (2022‐1‐8011).

## CONFLICT OF INTEREST STATEMENT

There is no conflict of interest to disclose. We confirm that we have read the journal's position on issues involved in ethical publication and affirm that this report is consistent with the guidelines.

## ETHICS STATEMENT

This study was retrospectively approved by the Ethics Committee of Sanbo Brain Hospital, Capital Medical University (SBNK‐2017‐15‐01).

## Supporting information


Appendix S1

## Data Availability

The data supporting this study's findings are available from the corresponding author upon reasonable request.
