# Supplementary material for: SEEG‐based reevaluation of epileptogenic networks and the predictive role for reoperation in MTLE patients with surgical failure
Source: Epilepsia Open. 2023 Apr 27;8(3):846–57. doi: 10.1002/epi4.12743 (PMC10472362; doi:10.1002/epi4.12743)
Supplement: Supplementary file 1 — Appendix S1 [file EPI4-8-846-s001.docx]

**2.4 SEEG analysis**

**2.4.1 SEEG data pre-processing**

Recordings of 2 minutes before and after the onset time-point, which are defined by two independent neuro-electrophysiologists, were selected for each epileptic seizure. The raw SEEG data was band-passed filtered (0.2-110Hz, basic FIR filter, below Nyquist frequency, before analyses), notched (50Hz), re-referenced with average, and channeled with artifacts removed in EEGLAB (https://sccn.ucsd.edu/eeglab/index.php, eeglab2022.0 toolbox). Referring to the implantation scheme of pre-surgical evaluation, electrode contacts within designed brain regions were selected to be merged to present time-array signals of seven brain regions: pHip, MTG, pSTG, ILG, ISG, OFG, and TPO. For each seizure onset, a piece of continuous 10-second segment from the onset time point was identified to explicitly present the information flow modality within the early period of seizure onset. The SOZ was defined as the depth electrode contacts showing the first unequivocal ictal intracranial EEG change. The early-propagation zone (EPZ) was defined as starting at least 500ms after seizure-onset and recorded outside the SOZ^1^. The SOZ and EPZ were both attributed to surgery zone (SZ), which was considered for reoperation. In the study, the connectivity indexes in the delta band (1-4Hz), theta band (4-8Hz), alpha band (8-13Hz), beta band (13-30Hz), low-gamma band (30-50Hz), the high-gamma band (50-80Hz) and ripple band (80-110Hz) were selected to analysis respectively. The 7 frequency bins according to different oscillation rhythms of seizure onset patterns^2^ were set to explore the effect of the oscillation band on the surgical prognosis. Before calculating FC, frequency transformation was performed using Fieldtrip Toolbox to identify the distribution of the power spectrum in each region. In total, 13 pieces, 10-second segments of 10 patients (favorable group: 7 seizures of 7 patients with Engel class I vs unfavorable group: 6 seizures of 3 patients with Engel class II or III) were included in the analysis to calculate FC indexes using MATLAB-based fieldtrip toolbox^3^ (http://www.fieldtriptoolbox.org/), Information Breakdown toolbox^4^ (ibTB, merged into fieldtrip toolbox), Multivariate Granger Causality toolbox^5^ (MVGC, http://www.sussex.ac.uk/sackler/mvgc), and Phase Slope Index toolbox^6^ (PSI, http://doc.ml.tu-berlin.de/causality/, merged into fieldtrip toolbox) respectively.

**2.4.2 Non-directed FC calculation**

Three non-directed measurements, including mutual information (MI) strength and imaginary coherence (icoh) within-brain region and between-brain regions, were calculated by the first 10-second SEEG segment after the seizure onset point. The MI was derived from information theory and used to quantify the number of interactions between neural populations^4^. The ibTB optimized algorithm for speed of calculation and implemented correction procedures to address a systematic error due to limited sampling. Single-trial SEEG recordings had been discretized into equally wide frequency bins to extract the power of distinct frequencies. This discretization data procedure was necessary because it was convenient to quantify the neural response as a discrete variable. The core function was entropy.m which allowed to compute the entropy-like quantities of building blocks, specifying direct estimation method, Panzeri & Treves bias correction procedure, discretization method of “eqpop”, and 10 bins across the frequency domain as recommended Fieldtrip default. The MI was calculated per electrode contact using full band frequency, then averaged across contacts within each region (MI_within_region_freq)^7^.

The simplest and most popular measure of interaction between SEEG contacts or brain regions was a generalization of coherence to specified frequency bins. Unlike complex coherency, the imaginary part of coherency was only sensitive to the synchronization of two sources which were time-lagged to each other, while it was insensitive to two signals derived from a common underlying source generator^8^. The conclusion was based on the fundamental assumption that an observed signal potential had no time-lag to underlying source activity, which was a widely accepted “quasi-static approximation” theory. It was feasible that the imaginary part was very small or even vanished if the time lag between the two sources was vanishing. Therefore, a non-vanishing imaginary part interpreted the true brain interaction. The imaginary part of complex coherency per electrode contact was extracted and averaged within each brain region as the “within-region” imaginary part of coherency respectively (icoh_within_region_freq). Similarly, the imaginary part of coherency in brain regions was calculated from the contacts in a given brain region to contacts in other areas sampled (icoh_region_flow_freq).

**2.4.3 Directed FC-Causality analysis**

Several methods had been proposed to estimate the direction of coupling and identify network leaders, referred to as causality analysis. One definition of causality has been formulated by Granger in econometrics^9^. Extensions of granger causality to multi-contact SEEG data have been proposed^10^. The Granger causality method was based on the linear assumption for the relationship between signals estimated by multivariate autoregressive (MVAR) models. It was also able to estimate directed information flow based on the nonparametric meansures^11^. The MVGC MATLAB Toolbox prediction of the Granger causality formalism was based on the vector autoregressive (VAR) modeling, including the regression order of the model, the autocovariance sequence, and the cross-power spectral density of the underlying process^5^. The Granger causality was calculated per brain region and then averaged among measures from a given region to all other regions, which was defined as the given region’s outward strength (granger_region_outward_freq). Similarly, a given region’s inward strength was defined as the average measures from all other regions to the given brain region (granger_region_inward_freq).

**2.4.4 Directed FC-phase slope index**

The phase slope index (PSI) estimated the direction of information flux in multivariate time series and distinguished the driver from the recipient^6^. The PSI index was highly robust against false estimates caused by confounding factors of background activity, which would be having nontrivial spectral properties and eventually be measured in unknown superposition in the signals. The proposed method was based on the slope of the phase spectrum of cross-spectra between two-time series, which was defined as channel_i and channel_j. Based on the assumption that interactions required some time, the phase differences between sender and recipient increased with frequency, thus resulting in a positive slope of the phase spectrum of cross-spectra, where cross-spectra was a contacts* frequency matrix. The positive slope of the phase spectrum was interpreted as the net information flux from channel_i to channel_j, and the negative slope was interpreted as the net information flux from channel_j to channel_i. It was insensitive to confound background activity regardless of spectral power and superpositions of activity in contacts. A pre-specified narrow band of 5 Hz width centered around the frequency spectral peak of interest, over which the slope was calculated. In comparison with Granger causality, the PSI index presented lower sensitivity to mixtures of independent background noise sources. The PSI Toolbox had been merged into the Fieldtrip toolbox, which was defined as ft_connectivity_psi.m function. The multivariate time series signals were processed with multi-taper frequency transformation based on the “mtmfft” method to get cross-spectra. The PSI index was calculated per brain region and averaged measures from a given region to all other regions, defined as region net information flux (psi_region_net_freq).

**Figure S1: The SEEG signals of two groups were merged to show the distribution of the power spectrum and the ROC curve of inner-region FCs modeling showed the prediction of surgical outcomes.**


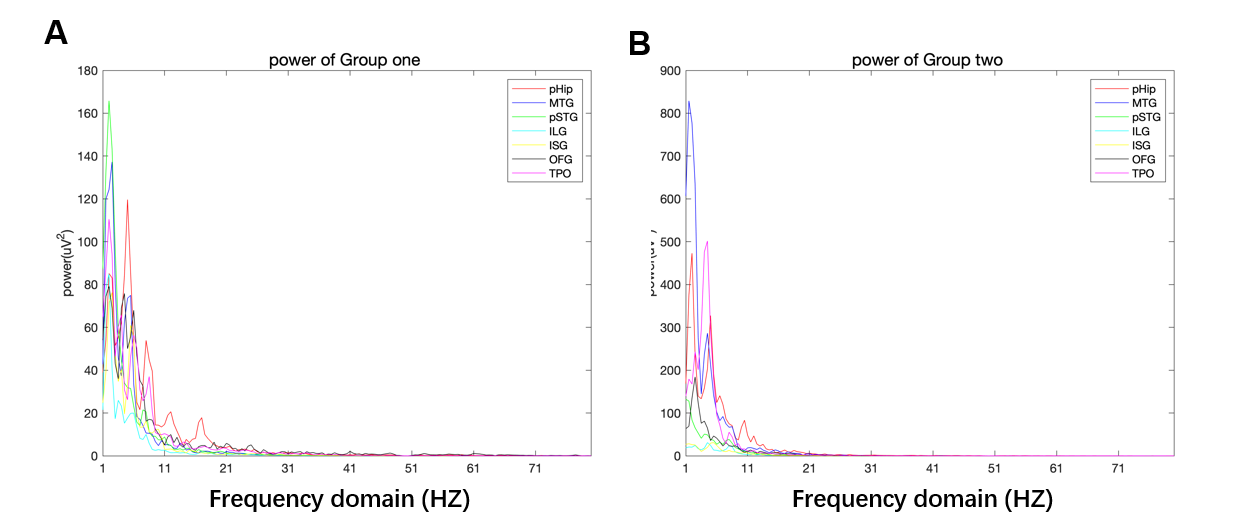


The power spectrum of group one **(A)** was significantly lower than that of group two **(B)**, especially showing a more oscillation power of low frequency.

**References**

1. Perucca P, Dubeau F, Gotman J. Intracranial electroencephalographic seizure-onset patterns: effect of underlying pathology. Brain. 2014;137(Pt 1):183-196.

2. Lagarde S, Buzori S, Trebuchon A, et al. The repertoire of seizure onset patterns in human focal epilepsies: Determinants and prognostic values. Epilepsia. 2019;60(1):85-95.

3. Oostenveld R, Fries P, Maris E, Schoffelen JM. FieldTrip: Open source software for advanced analysis of MEG, EEG, and invasive electrophysiological data. Computational intelligence and neuroscience. 2011;2011:156869.

4. Magri C, Whittingstall K, Singh V, Logothetis NK, Panzeri S. A toolbox for the fast information analysis of multiple-site LFP, EEG and spike train recordings. BMC neuroscience. 2009;10:81.

5. Barnett L, Seth AK. The MVGC multivariate Granger causality toolbox: a new approach to Granger-causal inference. Journal of neuroscience methods. 2014;223:50-68.

6. Nolte G, Ziehe A, Nikulin VV, et al. Robustly estimating the flow direction of information in complex physical systems. Physical review letters. 2008;100(23):234101.

7. Narasimhan S, Kundassery KB, Gupta K, et al. Seizure-onset regions demonstrate high inward directed connectivity during resting-state: An SEEG study in focal epilepsy. Epilepsia. 2020;61(11):2534-2544.

8. Nolte G, Bai O, Wheaton L, Mari Z, Vorbach S, Hallett M. Identifying true brain interaction from EEG data using the imaginary part of coherency. Clinical neurophysiology : official journal of the International Federation of Clinical Neurophysiology. 2004;115(10):2292-2307.

9. CWJ G. Some recent development in a concept of causality. J Econom 1988:12.

10. Cui J, Xu L, Bressler SL, Ding M, Liang H. BSMART: a Matlab/C toolbox for analysis of multichannel neural time series. Neural networks : the official journal of the International Neural Network Society. 2008;21(8):1094-1104.

11. Dhamala M, Rangarajan G, Ding M. Analyzing information flow in brain networks with nonparametric Granger causality. NeuroImage. 2008;41(2):354-362.
